# THE USE OF ABDOMINAL CORSETS ON COLONOSCOPY: A PROSPECTIVE RANDOMISED CONTROLLED TRIAL

**DOI:** 10.1590/0102-6720202500004e1873

**Published:** 2025-03-14

**Authors:** Mert ADALI

**Affiliations:** 1 Health Sciences University, Bursa Yuksek Ihtisas Training and Research Hospital, General Surgery Unit - Yildirim, Bursa, Turkey.

**Keywords:** Colonoscopy, Endoscopy, Bandages, Colonoscopia, Endoscopia, Bandagens

## Abstract

**BACKGROUND:**

Colonoscopy is a widely used endoscopic procedure to investigate diseases of the colon and rectum. Colonoscopy procedure has difficulties for the patient and endoscopist.

**AIMS:**

To investigate whether the use of an abdominal corset can make the colonoscopy procedure easier and faster.

**METHODS:**

This is a prospective randomised controlled study. Patients over 18 years of age who underwent elective colonoscopy in our clinic were evaluated. Patients were divided into two groups according to the use of the corset. Variables were compared between the groups.

**RESULTS:**

A total of 204 patients were included in the study. Corsets were used in 97 patients and not used in 107 patients. The need for manual compression was found to be decreased in the corset use group. There was no effect of corset use on cecal intubation time in the general population. It was found that cecal intubation time decreased with corset use in patients with body mass index - BMI<30 and male gender.

**CONCLUSIONS:**

The need for manual compression can be reduced by the use of an abdominal corset during colonoscopy. The use of an abdominal corset may make the colonoscopy procedure faster and easier for the endoscopist and the patient.

## RESUMO

**RACIONAL:**

A colonoscopia é um procedimento endoscópico amplamente utilizado para investigar doenças do cólon e do reto. O procedimento de colonoscopia apresenta dificuldades para o paciente e o endoscopista.

**OBJETIVOS:**

Investigar se o uso de um espartilho abdominal pode tornar o procedimento de colonoscopia mais fácil e rápido.

**MÉTODOS:**

Este é um estudo prospectivo controlado e randomizado. Foram avaliados pacientes com mais de 18 anos de idade que se submeteram à colonoscopia eletiva em nossa clínica. Os pacientes foram divididos em dois grupos de acordo com o uso do espartilho. As variáveis foram comparadas entre os grupos.

**RESULTADOS:**

O total de 204 pacientes foram incluídos no estudo. Os espartilhos foram usados em 97 pacientes e não foram usados em 107 pacientes. Verificou-se que a necessidade de compressão manual diminuiu no grupo que usou o espartilho. Não houve efeito do uso do espartilho no tempo de intubação cecal na população em geral. Verificou-se que o tempo de intubação cecal diminuiu com o uso do espartilho em pacientes com IMC<30 e do sexo masculino.

**CONCLUSÕES:**

A necessidade de compressão manual pode ser reduzida com o uso de um espartilho abdominal durante a colonoscopia. O uso de um espartilho abdominal pode tornar o procedimento de colonoscopia mais rápido e fácil para o endoscopista e o paciente.

## INTRODUCTION

Colonoscopy is an endoscopic procedure commonly used for screening, diagnosis and treatment. Today, it has become a routine medical procedure. It is a safe and effective method of examining the colon and rectum. However, colonoscopy can be a challenging procedure for both patients and colonoscopists. A difficult colonoscopy usually takes a long time and is associated with increased complication rates. Sigmoid loops cause severe pain and can increase the risk of associated complications such as bowel perforation or splenic injury^4^ ^,^ ^9^ ^,^ ^11^. Auxiliary manoeuvres such as position change and manual compression are often used to prevent looping. When patients are sedated, position change is a difficult manoeuvre to perform for the patient and staff. Manual compression may cause uncontrollable pressure and depends on the skill of the assistants performing it. The use of an abdominal corset during the procedure can provide balanced and effective pressure. Several randomised controlled trials (RCTs) have investigated the efficacy of abdominal compression devices in colonoscopies^3^ ^,^ ^7^ ^,^ ^12^.

The hypothesis of this study was that the use of an abdominal corset may reduce looping in patients undergoing colonoscopy. Therefore, a prospective study was conducted to investigate whether the use of an abdominal corset could make the colonoscopy procedure easier and faster for the patient and colonoscopist.

## METHODS

### Study design

This is a prospective, randomised controlled trial. Approval was obtained from Health Sciences University Bursa High Speciality Training and Research Hospital Clinical Research Ethics Committee (number 2011-KAEK-25 2023/02-08, 08/02/2022). From March 2023 to December 2023, 204 patients who underwent colonoscopy were included in the study. All colonoscopies were performed by the same endoscopist. All patients included in the study were informed before the procedure and gave their informed consent.

### Study population

Patients over 18 years of age, scheduled for elective colonoscopy and who had completed bowel cleansing were included in the study. Patients with inadequate bowel cleansing, history of previous colorectal surgery, therapeutic intervention during the procedure, detection of a mass and incomplete colonoscopy were excluded from the study.

### Data collection and colonoscopy procedure

Eligible patients were randomised to the corset or non-corset group using a computer-generated random number list. Data were collected in the corset and non-corset groups. The abdominal corset used was an elastic fabric corset with adjustable width to fit all patients. Bowel preparation was performed with a rectal enema containing sodium phosphate and laxatives. All patients were sedated by the anaesthesia team. Patients were placed in the left lateral decubitus position.

Age, weight, height, sex, history of abdominal surgery, corset use, cecal insertion time (CIT), manual compression or position change, and Boston Bowel Preparation Scale (BBPS) data were recorded by the endoscopist. The colonoscope insertion time from anus to cecum was recorded and rounded to the nearest minute.

### Statistical analysis

IBM Statistical Package for the Social Sciences (SPSS) Statistics for Windows, version 21.0 (IBM Corp. Armonk, NY: USA. Released 2012) was used for statistical analyses. Descriptive statistics were expressed as mean±standard deviation (minimum-maximum), median and range and/or interquartile range (IQR) for numerical variables, while categorical variables were expressed as number of cases and (%). The Kolmogorov-Smirnov test was used to test the normality of the data distribution. Levene’s test was used to determine whether the assumption of homogeneity of variances was met. The Mann-Whitney U test was used to assess the significance of the difference between groups for continuous numerical variables for which the statistical assumptions of parametric testing were not met. χ^2^ and Fisher’s exact test were used to analyse the relationship between categorical variables. p<0.05 was considered statistically significant. Results were presented with 95% confidence interval.

## RESULTS

**Figure 1:**
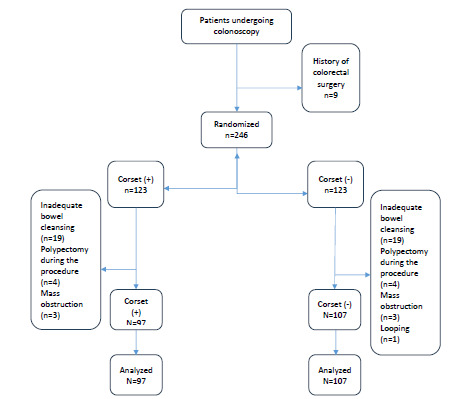
Flow diagram.

A total of 255 patients underwent colonoscopy under sedation, in the same period of this study; however, 51 patients were excluded for various reasons. Thirty-one patients were excluded due to inadequate bowel preparation, nine patients due to a history of previous colorectal surgery, six patients due to therapeutic intervention (polypectomy) during the procedure, four patients due to mass obstruction and one patient due to looping. Therefore, a total of 204 patients were included in the study (Figure 1).

Of the patients included in the study, 87 were male (42.6%) and 117 were female (57.4%). The median age was 57 years. An abdominal corset was used in 97 patients (47.5%) and the procedure was performed without a corset in 107 patients (52.5%). The median cecal intubation time was 4.5 minutes. The clinical characteristics of the patients are presented in Table 1.

**Table 1:** Distribution of clinical and demographic characteristics.

Gender	
Male	87 (42.6)
Female	117 (57.4)
Comorbidities	
HT	60 (29.4)
DM	45 (22.1)
CHD	25 (12.3)
COPD	12 (5.9)
Other	40 (19.6)
Indication	
Anaemia	32 (15.7)
FOBT (+)	32 (15.7)
Constipation	43 (21.1)
Scanning	53 (26)
Haematochezia	22 (10.8)
History of polyps	10 (4.9)
CT suspicion	6 (2.9)
Chronic diarrhoea	6 (2.9)
History of Abdominal Surgery	
Yes	84 (41.2)
No	120 (58.8)
Type of surgery	
TAH-BSO	22 (10.8)
C/S	22 (10.8)
Appendectomy	22 (10.8)
Cholecystectomy	21 (10.3)
Gastrectomy	6 (2.9)
Other	10 (4.9)
Manual compression	
Yes	41 (20.1)
No	163 (79.9)
Change of position	
Yes	1 (0.5)
No	203 (99.5)
Corset	
Yes	97 (47.5)
No	107 (52.5)
Diagnosis	
Normal	148 (72.5)
Polyp	34 (16.7)
Diverticulum	13 (6.4)
Colitis	5 (2.5)
Mass	2 (1)
Parasite	2 (1)
Age, median IQR (25-75)	57 (47-65)
Weight, mean±SD	76.9±13.4
Height, median IQR (25-75)	165 (160-172)
BMI mean±SD	27.9±4.5
BBPS, median IQR (25-75)	8 (7-9)
Cecal intubation time, median IQR (25-75)	4.5 (3-6.5)

HT: hypertension; DM: diabetes mellitus; CHD: chronic heart disease; COPD: chronic obstructive pulmonary disease; FOBT: fecal occult blood test; CT: computer tomography; TAH-BSO: total abdominal hysterectomy with bilateral salpingo-oophorectomy; C/S: cesarean section; IQR: interquartile range; SD: standard deviation; BMI: body mass index; BBPS: Boston bowel preparation scale.

Weight, BMI, age, height, BBPS and gender did not show a statistically significant difference with or without the use of an abdominal corset (p>0.005). When comparing the two groups, the use of the corset was found to have no significant effect on cecal intubation time (p>0.05). Abdominal corset use was found to statistically and significantly reduce the need for manual compression (p<0.05) (Table 2).

**Table 2:** Comparison of variables according to corset use.

Variables	Corset (+) (n=97)	Corset (-) (n=107)	p-value
Weight	78.2 ± 13.7	75.7 ± 13	0.186*
BMI	28.1 ± 4.7	27.7 ± 4.4	0.532*
Age	57 (43-65)	57 (50-66)	0.407^†^
Height	165 (160-173)	165 (160-170)	0.248^†^
BBPS	8 (7-9)	8 (7-9)	0.272^†^
Cecal intubation time	4 (3-6)	4.5 (3.5-7)	0.137^†^
Gender			
Male	45 (46.4)	42 (39.3)	0.375^‡^
Female	52 (53.6)	65 (60.7)	
History of Abdominal Surgery			
Yes	42 (43.3)	42 (43.3)	0.657^‡^
No	55 (56.7)	65 (60.7)	
Manual compression			
Yes	12 (12.4)	29 (27.1)	0.014^‡^
No	85 (87.6)	78 (72.9)	
Change of position			
Yes	0	1 (0.9)	0.525^§^
No	97 (100)	106 ( 99.1)	

*independent samples t-test; ^†^Mann-Whitney U; ^‡^Chi-square test; ^§^Fisher’s Exact Test. BMI: body mass index; BBPS: Boston bowel preparation scale.

Male and female patients were analysed separately according to the use of corsets. While the use of an abdominal corset had no effect on cecal intubation time in female patients, it statistically significantly decreased cecal intubation time in male patients (p<0.05) (Table 3).

**Table 3:** Evaluation of corset use by male and female gender separately.

Variables	Male (n=87)			Female (n=117)		
	Corset (+) (n=45)	Corset (+) (n=42)	p-value	Corset (+) (n=52)	Corset (+) (n=65)	p-value
Age	53±14	58±12.9	0.102*	54±12.9	54±11,2	0,756*
Weight	81±13.3	80±10.4	0.683^†^	75±13.5	72±13,7	0,290^†^
Height	174±6.1	173±5.7	0.662*	158±14.2	159±5	0,771*
BMI	26.7±3.7	26.6±3.6	0.881^†^	29.3±5.1	28,4±4,8	0,339^†^
BBPS	8±0.9	7±1	0.353*	8±0.9	7,9±1	0,487*
Cecal intubation time	4±1.7	4.8±2	0.036*	5.5±2.8	5,4±2,6	0,891*
	4.4±1.9			5.4±2.7		0.003*

*Mann-Whitney U; ^†^independent samples t-test.

BMI: body mass index; BBPS: Boston bowel preparation scale.

Patients were evaluated in two groups, BMI<30 and BMI=30. Corset use was found to statistically and significantly reduce cecal intubation time in the BMI<30 group. In the BMI=30 group, the effect of corset use on cecal intubation time was not statistically significant (Table 4).

**Table 4:** Comparison of corset use grouped according to body mass index.

Variables	BMI<30 (n=150)			BMI=30 (n=54)		
	Corset (+) (n=45)	Corset (-) (n=42)	p-value	Corset (+) (n=52)	Corset (-) (n=65)	p-value
Cecal intubation time	4.5±2.1	5.1±2.3	0.025*	5.6±3.2	5.2±2.6	0.794*
	4.8±2.2			5.4±2.9		0.367*
Manual compression						
Var	8 (11.1)	23 (29.5)	0.010^†^	4 (16)	6 (20.7)	0.927^†^
Yok	64 (88.9)	55 ( 70.5)		21 (84)	23 (79.3)	

*Mann-Whitney U=χ^2^ test; ^†^independent samples t test.

BMI: body mass index.

## DISCUSSION

This study investigated the effects of abdominal corset use during colonoscopy in the general population. No statistically significant effect of corset use on cecal intubation time was found (p>0.05), but it was observed to decrease the need for manual compression (p<0.05). The results obtained in a similar study using an abdominal compression device were similar to our findings^3^. The decrease in the need for manual compression indicates that the use of an abdominal corset may replace assistant-dependent and uncontrollable manoeuvres. In the study of Toros et al.^12^, it was observed that the use of an abdominal corset decreased the duration of cecal intubation and the need for manual compression. In another study, recently, the authors also confirmed that the use of an abdominal corset reduced the duration of cecal intubation and the need for manual compression^13^. The difference in cecal intubation time from similar studies is thought to be due to the fact that the procedure is an endoscopist-dependent procedure.

Male and female gender were evaluated separately according to corset use. It was found that corset use statistically and significantly reduced cecal intubation time in males (p<0.05). No difference was observed in females. The reason for this difference may be related to the anatomical difference between men and women. It has been suggested that the length of the sigmoid colon and the height of the sigmoid mesentery are greater in men, and therefore looping is more common. In a cadaveric study, the length of sigmoid colon and sigmoid mesocolon were found to be significantly longer in males than females^8^. In a study investigating the role of anatomical dimensions of the sigmoid colon in the development of sigmoid volvulus, it was found that sigmoid colon length and sigmoid mesentery height were significantly longer in men^2^.

In studies comparing male and female sex with regard to cecal intubation times, the results are different. While one study found that the cecal intubation time was longer in women^5^, another found it to be longer in men^1^. In our study, cecal intubation time was found to be statistically significantly shorter in males than females (p<0.05). It is known that the length of the colon is longer in women than in men^10^. Although the duration of colonoscopy is shorter in the female gender with a longer colon, the use of a corset is an effective method to reduce the duration of cecal intubation in the male gender with a longer sigmoid colon and higher sigmoid mesentery.

There are studies showing that an increased BMI is associated with prolonged cecal intubation time^6^. In our study, no association was found between BMI and cecal intubation time (p>0.05). When we divided the patients into two groups according to BMI and compared the use of corsets, we obtained statistically significant results. It was found that corset use reduced cecal intubation time in the group with BMI<30 (p<0.05). Liu et al.^7^ reported, in a prospective randomised study, that abdominal bandages significantly reduced cecal intubation time in obese patients. There may be many reasons for the lack of effect of the use of abdominal corsets on cecal intubation time in obese patients in our study. One of these reasons is that the compression effect of the abdominal corset reduces the function of preventing looping due to the increase in subcutaneous adipose tissue thickness. It is thought that the compression effect of the abdominal corset is better in the BMI<30 group of patients, thus reducing the cecal intubation time.

This is a prospective study in which all data were collected regularly and systematically. All colonoscopies were performed by a single endoscopist. In addition to studies supporting the use of an abdominal corset in large populations, this study recommends the use of abdominal corsets in specific populations (male, BMI<30). The study has some limitations. It is a single-centre study. The tightness of the corset was not consistent in all patients because it depended on the patient’s comfort.

## CONCLUSIONS

The use of an abdominal corset during colonoscopy reduces the need for manual compression. It reduces cecal intubation time in male patients and in patients with BMI<30. Multicentre, randomised controlled trials on this subject are needed.

## Central Message

Today, colonoscopy has become a routine medical procedure. However, it can be a challenging procedure for both patients and colonoscopists. A difficult colonoscopy usually takes a long time and is associated with increased complication rates. Auxiliary manoeuvres such as position change and manual compression are often used to prevent looping. When patients are sedated, position change is a difficult manoeuvre to perform for the patient and staff. Manual compression may cause uncontrollable pressure and depends on the skill of the assistants performing it.

## Perspectives

This study investigated the effects of abdominal corset use during colonoscopy in the general population. The use of an abdominal corset during colonoscopy reduces the need for manual compression. It reduces cecal intubation time in male patients and in patients with body mass index - BMI<30. Therefore, the use of an abdominal corset may make the colonoscopy procedure faster and easier for the endoscopist and the patient.

## References

[1] Akere A, Otegbayo JA (2016). Complete colonoscopy impact of patients' demographics and anthropometry on caecal intubation time. BMJ Open Gastroenterol.

[2] Atamanalp SS, Öztürk G, Aydinli B, Ören D (2011). The relationship of the anatomical dimensions of the sigmoid colon with sigmoid volvulus. Turk J Med Sci.

[3] Crockett SD, Cirri HO, Kelapure R, Galanko JA, Martin CF, Dellon ES (2016). Use of an abdominal compression device in colonoscopy a randomized, sham-controlled trial. Clin Gastroenterol Hepatol.

[4] Hsieh YH, Tseng KC, Chou AL (2010). Patient self-administered abdominal pressure to reduce loop formation during minimally sedated colonoscopy. Dig Dis Sci.

[5] Hwang YJ, Shin DW, Kim N, Yoon H, Shin CM, Park YS (2021). Sex difference in bowel preparation quality and colonoscopy time. Korean J Intern Med.

[6] Krishnan P, Sofi AA, Dempsey R, Alaradi O, Nawras A (2012). Body mass index predicts cecal insertion time the higher, the better. Dig Endosc.

[7] Liu TT, Meng YT, Xiong F, Wei C, Luo S, Zhan SG (2022). Impact of an abdominal compression bandage on the completion of colonoscopy for obese adults a prospective randomized controlled trial. Can J Gastroenterol Hepatol.

[8] Michael SA, Rabi S (2015). Morphology of sigmoid colon in south indian population a cadaveric study. J Clin Diagn Res.

[9] Moura DTH, Baroni LM, Bestetti AM, Funari MP, Rocha RSP, Santos MEL (2024). Evaluation of quality indicators of screening colonoscopy performed in a private quarternary hospital in Brazil. Arq Bras Cir Dig.

[10] Saunders BP, Fukumoto M, Halligan S, Jobling C, Moussa ME, Bartram CI (1996). Why is colonoscopy more difficult in women. Gastrointest Endosc.

[11] Shah SG, Saunders BP, Brooker JC, Williams CB (2000). Magnetic imaging of colonoscopy an audit of looping, accuracy and ancillary maneuvers. Gastrointest Endosc.

[12] Toros AB, Ersoz F, Ozcan O (2012). Does a fitted abdominal corset makes colonoscopy more tolerable. Dig Endosc.

[13] Yu GQ, Huang XM, Li HY, Tang W, Hu DM, Lü MH (2018). Use of an abdominal obstetric binder in colonoscopy a randomized, prospective trial. J Gastroenterol Hepatol.

